# Chronic Exposure to Organophosphates Pesticides and Risk of Metabolic Disorder in Cohort from Pakistan and Cameroon

**DOI:** 10.3390/ijerph18052310

**Published:** 2021-02-26

**Authors:** Mbah Ntepe Leonel Javeres, Rabia Habib, Ngondi Judith Laure, Syed Tahir Abbas Shah, Martin Valis, Kamil Kuca, Syed Muhammad Nurulain

**Affiliations:** 1 Department of Biosciences, COMSATS University Islamabad, Chak Shahzad, Islamabad 45550, Pakistan; mbahjl@yahoo.fr (M.N.L.J.); rabiahabib@comsats.edu.pk (R.H.); syedtahirabbas@comsats.edu.pk (S.T.A.S.); 2 Institute of Medical Research and Medical Plants Studies, Yaounde 1457, Cameroon; 3 Department of Biochemistry, Yaoundé I University, Yaoundé 8024, Cameroon; ngondijudithl@hotmail.com; 4 Department of Neurology of the Medical Faculty of Charles University and University Hospital in Hradec Kralove, Sokolska 581, 50005 Hradec Kralove, Czech Republic; martin.valis@fnhk.cz; 5 Biomedical Research Center, University Hospital in Hradec Kralove, Sokolska 581, 50005 Hradec Kralove, Czech Republic; 6 Department of Chemistry, Faculty of Science, University of Hradec Kralove, 50003 Hradec Kralove, Czech Republic

**Keywords:** metabolic disorder, pesticides, organophosphorus, dyslipidemia, liver malfunctioning

## Abstract

(1) Background: Organophosphorus pesticides (OPPs) are major chemicals used in agriculture for eradication of insecticides/pesticides. Unfortunately, the longtime exposure of human beings to OPPs could lead to metabolic disorder such as high blood pressure, hyperglycemia, overweight or dyslipidemia. The aim of this research is to evaluate the possible metabolic dysregulations as a consequence of chronic OPPs exposure to individuals in Cameroon and Pakistan. (2) Methods: Blood samples were collected from 300 participants in each country, into ethylenediaminetetraacetic acid (EDTA) tubes. The samples were extracted with solid phase extraction (methanol/water) for analysis of OPPs with gas chromatography mass spectrometry. The spectrophotometry and Enzyme Linked ImmunoSorbent Assay (ELISA) were used to measure the hepatic, renal, pancreatic and cardiovascular functions. The atherogenic index (AI) was also determined in OPPs exposed and nonexposed cohorts. (3) Results: The results showed the presence of malathion, parathion and chlorpyrifos OPPs residues in Cameroonians, and malathion and chlorpyrifos in Pakistani samples, respectively. Elevated Body Mass Index (BMI), insulin, blood glucose, dyslipidemia and hypertension were noted in OPPs chronic exposed groups. In addition, dysregulated liver and kidney function profiles were observed in all participants regardless of gender and age groups. (4) Conclusions: The study concludes that both the study cohorts showed several metabolic dysregulations attributable to chronic exposure to a mixture of OPPs which may provide precursors for establishment of metabolic syndrome and other chronic diseases. Further different extended population-based studies are suggested to understand the differential metabolic dysfunctions caused by structurally different OPPs mixtures exposure.

## 1. Introduction

Development of metabolic diseases as a consequence of exposure to pesticides has been extensively reported in the literature [1]. Pesticides are hazardous for human health because they can impair metabolic homeostasis [2]. Epidemiological studies have provided evidence for linkage of pesticide exposure with endocrine disruption, insulin resistance, nonalcoholic fatty liver disease, obesity, diabetes and so forth [3,4]. Of these metabolic diseases, metabolic syndrome (MetS) refers to a combination of interdependent metabolic abnormalities whose clinical significance and exact etiology remains elusive. This syndrome is clinically diagnosed when the subject has at least three of the following symptoms: high blood pressure, high blood sugar, overweight or dyslipidemia. The combination of these symptoms can lead to complications such as heart disease, stroke, and liver and/or kidney disease [5]. The prevalence of MetS has been significantly increasing worldwide over the past decades [5]. For example, in the United States, the prevalence of MetS has increased from 32.9% in 2004 to 34.7% in 2015; in Korea from 24.9% in 1998 to 31.3% in 2007; and in Malaysia, where the last recorded prevalence of metabolic syndrome was 42.5% in 2013 [6]. Similarly, in Pakistan, more than 18% of the population suffers from metabolic diseases [7]. In sub-Saharan Africa, it was projected that MetS affected around 4.5% of the population in 2011 and that is projected to increase to 19% by 2035 [8]. Multiple factors have been shown to be responsible for the appearance and progression of these pathologies, which can be either of endogenous origin such as susceptibility genes, or exogenous, that is, a set of environmental factors. Among the environmental factors, pesticides represent significant environmental contaminants and are the cause of a significant amount of mortality and morbidity in areas of high exposure [9]. Epidemiological studies also show that the prevalence of cancer, birth defects, infertility problems, neurological problems or a weakened immune system are significantly increasing in pesticide-exposed individuals [1]. Organophosphorus compounds are heavily used as pesticides (about 50 percent of the global use) and are expected to increase considerably by 2030, which in turn will pose a risk of direct health consequences [10,11]. The harmful effects of several organophosphorus pesticides on the etiology of metabolic diseases have been demonstrated by a growing number of studies involving both humans and laboratory animals [12,13]. The results of those studies have often come up with controversial results; for example, Li et al. [1] showed that exposure to OPPs (organophosphate pesticides) increases several biochemical parameters including glucose, LDL cholesterol, insulin, HbA1c and HOMA-IR, predisposing individuals to serious metabolic diseases such as diabetes, cardiovascular disease, kidney disease, stroke, liver disease and atherosclerosis. However, other studies showed that despite OPPs exposure, the biochemical parameters remained unchanged (normoglycemia [14], normo-insulinemia [15]) or decreased (hypoglycemia [16], hypoinsulinemia [14]).

It is noteworthy that most of the studies on metabolic diseases are in context with acute exposure, mostly to the organochlorine group of pesticides. Almost negligible reports may be traced to the incidence of metabolic diseases in the context of chronic exposure of the mixture of organophosphates. The present work has studied the possible metabolic dysregulation due to chronic exposure of two or more OPPs pesticides on distinct cohorts of Pakistani and Cameroonian origin. To the best of our knowledge, this is the first such study with geographically and genetically distinct populations in connection with chronic exposure to OPPs and development of metabolic disorder.

## 2. Materials and Methods

### 2.1. Study Design and Sampling

A cross-sectional study with random sampling from known pesticide-exposed agriculture areas was selected. Across the board, the study participants were recruited from cotton farming areas where organophosphates are mainly applied. In Cameroon, the agricultural towns located in the North (Mora and Figuil) and in the South (Njobe and Sa’a) were selected on this basis. In Pakistan, the cities of Depalpur and Multan were selected. Ethical approvals, CIIT/BIO/ERB/19/90 and No. 488/CE/CNERSH were obtained from the ethical review board of CUI, Islamabad and Cameroon, respectively, for the study. The study followed the principles of the Declaration of Helsinki on human trials. Research participants were informed about the goal of the study and permissions were taken in writing before sample collections. The age group of recruited volunteers was 18 to 40 years. In addition, they were inhabitants of the pesticide-sprayed areas for at least six months and were exposed to OPPs directly (farmers and sprayers) or indirectly (people living in the spraying areas) during the period. Subject volunteers using products other than OPPs and those suffering from diseases such as diabetes, neurological diseases, cancers and all chronic pathologies were excluded from the study.

The participants whose blood tests showed the presence of pesticides other than OPPs were also excluded from the study. Samples from unexposed subjects were collected in nonagricultural areas with less risk of exposure to pesticides (thus limiting indirect exposure).

Sociodemographic characteristics and parameters such as age, sex, height, weight, tobacco/narcotic consumption, history of exposure, use of protective equipment, duration of activity, and professional data were collected using a questionnaire. MetS has been diagnosed when the subject exhibits at least three of the following symptoms: high blood pressure, high blood sugar, overweight or dyslipidemia, as previously reported [5].

Using convenient nonprobability sampling, 300 blood samples were collected from exposed persons in each country. The sample size was calculated using the Lorenz formula for a cross-sectional study according to the prevalence of metabolic disease in the Cameroonian and Pakistani populations.

Blood samples were taken once from all participants by venipuncture and transported to the laboratory using an icebox. In the laboratory, the blood was centrifuged at 3500 rpm for 10 min at 25 °C, and the resulting plasma was then stored at −80 °C until further analysis. The samples were analyzed at the IMPM laboratory, Yaoundé, Cameroon, and in the laboratories of the Department of Biosciences of COMSATS Islamabad University, Pakistan.

### 2.2. Assesment of Pesticide Residues

Pesticides were screened in plasma by gas chromatography coupled with high resolution mass spectrometry (GC-MS System 5975C Agilent, Milton Keynes U.K.) as described in [17]. The types of pesticides were further confirmed from farmers (Cotton), the pesticides in stock, and after the inspection of the fields and dumping points of abandoned pesticide packages in villages. The column was calibrated at 120 °C for 1 min, then set to increase its temperature up to 290 °C. The temperatures of the injection orifice and the interface were stabilized at 250 °C, and the temperature of the detector at 290 °C. The injection mode without division and helium with a flow rate of 1.0 mL/min were used as carrier gas. Calibration assay and internal quality control were performed by addition of known concentrations of commercial solutions of chlorpyrifos, malaoxon, parathion, and internal standards (azobenzene) to drug-free whole blood. The six concentrations used for calibration curves of chlorpyrifos were 0.15, 0.5, 1.0, 1.25, 2.5 and 5.0 mg/L. In the case of malaoxon and parathion, these were 0.17, 0.5, 1.0, 1.25, 2.5, 5.00 mg/L and 0.13, 0.5, 1.0, 1.25, 2.5, 5.00 mg/L, respectively. The concentration of internal standard was 0.25 mg/L. Electron impact (EI) mass spectra of pesticides and internal standard were recorded by total ion monitoring. For quantification, the surface/peak ratios of the target ion of different insecticides and azobenzene (*m*/*z* 182) were calculated as a function of the concentration of the substance. The data were recorded only when the peak was clearly visible, and the signal-to-noise ratio was greater than three [18]. Limit of detection (LOD) and limit of quantification (LOQ) were estimated for the validation of this method. LOD and LOQ were determined by analyzing drug-free blood samples fortified with known drug concentrations. Each concentration was measured in five replicates. LOD was defined as the lowest concentration giving a response of three times the average baseline noise defined from five unfortified samples. LOQ was defined as the lowest observed concentration within 10% of the theoretical concentration with acceptable qualifier ion ratios for all five replicates. Ten blank blood samples were analyzed for chromatographic interference with each analyte.

### 2.3. Cholinesterase Activity

Organophosphates exposure was also assessed by measuring cholinesterases, that is red blood cell-acetylcholinesterase (RBC-AChE) and butyrylcholinesterase (BChE). RBC-AChE and BChE activity were determined using Ellamn’s method modified by Worek et al. [19] using Specord 50 plus spectrophotometer Number 233H1280C (Analytic Jena, Thuringia, Germany). RBC-AChE was measured from whole blood, and plasma was taken for BChE. Blood and plasma dilutions were prepared according to the method described by Worek et al. [19]. Spectrophotometric measurement was taken at 436 nm (ε = 10.6 × 10^3^ M^−1^ cm^−1^) at 37 °C for AChE and BChE. For hemoglobin, absorption was noted at 546 nm (ε = 10.8 × 10^3^ M^−1^ cm^−1^).

### 2.4. Biochemical Assesment

Plasma glucose was determined by an enzymatic and colorimetric method using glucose oxidase (GOD) according to the manufacturer’s protocols (Biolabo Kit 80009, from BIOLABO SA, Les Hautes Rives, 02160, Maizy, France). Creatinin (using Biolabo kit 80107), Blood urea nitrogen (using Biolabo kit 80221), Aspartate transaminase (using Biolabo kit 80025), Alanine transaminase (using Biolabo kit 80027), Gamma-glutamyl-transpeptidase (γGT) (using Biolabo kit 81110), Alkaline phosphatase (using Biolabo kit 92214), Lactate dehydrogenase (using Biolabo kit 92111), Bilirubin total and direct (using Biolabo kit 80403), and HDL (using Biolabo Kit 90206) were determined by enzymatic assays according to the manufacturer’s protocols provided by the kits of BIOLABO SA. The triglycerides were also determined in plasma by an enzymatic and colorimetric method using the Biolabo kit from Biolabo.

LDL Cholesterol and the atherogenic index (AI) were determined according to the formula provided by HDL kit:

LDL = Total cholesterol – TG/5 – HDL.

AI = LDL Cholesterol/HDL Cholesterol [20].

The Lipoprotein a Lp (a) was determined using an Enzyme-Linked Immunosorbent Assay (ELISA) kit (IBL-RE59011, IBL, Hamburg, Germany) according to the manufacturer’s protocol. Insulin was also determined by the ELISA assay (IBL-Kit RE 53171. Paraoxonase 1 (PON1; kit E-EL, Houston, TX, USA) was determined by the ELISA technique according to the manufacturer’s protocol.

### 2.5. Statistical Analysis

Statistical analysis of the data was performed using Stata 15 and R 3.2.0 software. Regression and correlation models were used to establish a linear relationship between dependent and independent variables. The Shapiro–Wilk test was used to check the normality of the distribution of variables. A one-way ANOVA test and post hoc tests were performed where needed. Odds ratios (OR) with 95% confidence intervals (95% CI) were calculated to establish the margins of significance. The differences were considered statistically significant if *p* value < 0.05.

## 3. Results

### 3.1. Socio-Demographic and Clinical Characteristics of OPPss Exposed Study Subjects

The distribution of the population by socio-demographic characteristics shows that the average age was 26.57 years in Cameroon against 28.22 years in Pakistan. The sex ratio (Male/Female) was 2.16 in favor of men in the Pakistani cohort against 0.90 in the Cameroonian group. The most represented level of education was the primary level among Pakistanis and the secondary/university level among Cameroonians. The variation of anthropometric and clinical parameters as a function of exposure (Table 1) reveals that in the exposed group, 40.45% of Pakistan’s population were overweight (BMI ≥ 25), 39.09% were obese, 62.27% had high blood pressure, and 12.73% take tobacco at a regular frequency. In the exposed population of Cameroon, 38.64% were overweight and 40.91% were obese, 76.36% had high blood pressure, and 4.54% had a high frequency of smoking.

### 3.2. OPPs Exposure Markers

**Figure 1:**
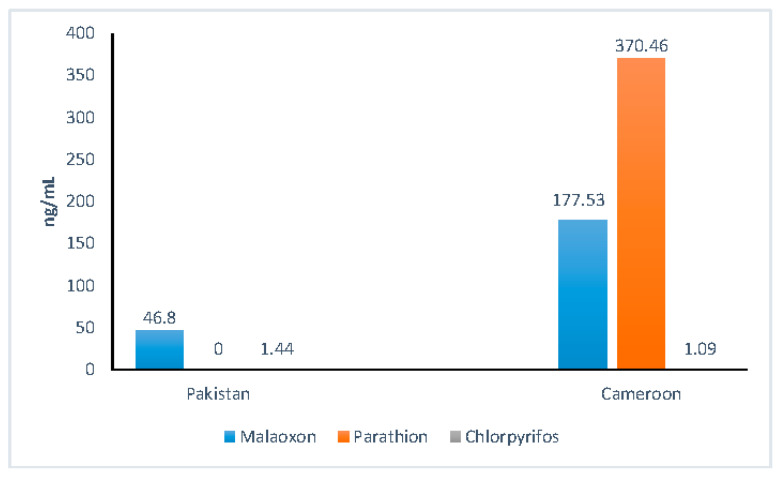
Distribution of blood pesticides levels in Pakistani and Cameroonian groups.

OPPs exposure markers, that is AChE and BChE, were decreased in exposed individuals in both countries with inhibition of 62.5% for Pakistanis and 54.54% for Cameroonians. Inhibition was more pronounced in the Pakistanis group. Table 2 shows the variation of AChE, BChE and paraoxonase (PON-1) in the two population groups. A significant decrease of PON-1 enzyme was observed in both populations groups. The determination of OPPs residues by GC-MS (Figure 1) revealed the presence of three OPPs: malathion (177.53 ± 99.74 ng/mL), parathion (370.46 ± 129.81 ng/mL) and chlorpyrifos (1.09 ± 0.44 ng/mL) in Cameroonian OPPs-exposed individuals. In the Pakistani exposed population, only malathion and chlorpyrifos were detected with values of 48.80 ± 30.12 ng/mL and 1.44 ± 0.98 ng/mL, respectively. Apart from biochemical confirmation from blood samples of the participants, agriculture areas were visited, and the empty containers of pesticides used in the area were inspected and verbally confirmed by the spraying personnel.

### 3.3. Biochemical Tests: Lipid Profile

**Figure 2:**
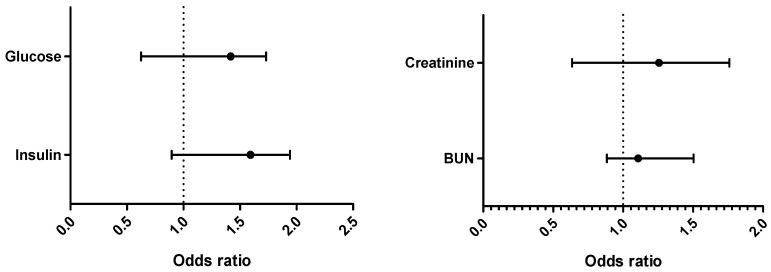

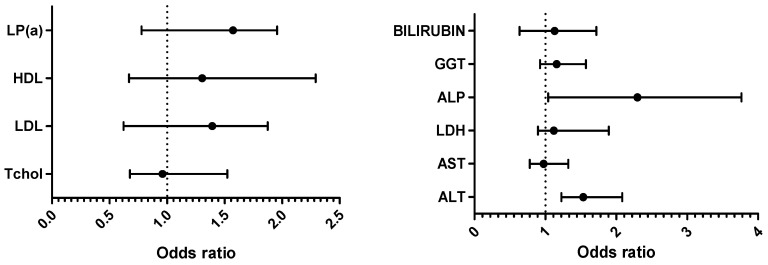
Binomial logistic regression (BLR) of all the biochemical parameters: Exposed Pakistan vs. exposed Cameroon (Tchosl = total cholesterol; HDL = high density lipoprotein; LDL = low density lipoprotein; Lp(a) = Lipoparticule a; AI = Arterogenic Index; BUN = Blood urea nitrogen; AST = aspartate transaminase; ALT = alanine transaminase; LDH = lactate deshydrogenase; GGT = gamma-glutamyl transferase; ALP = alkalin phosphatase; DBil = direct bilirubin; TBil = total bilirubin).

Variations in the biochemical parameters are shown in Table 3. All the tested lipid profile parameters were significantly affected in OPPs-exposed groups with metabolic syndrome risks. However, HDL was significantly different in the Cameroonian group. Logistic regression of biochemical parameters in exposed populations (Figure 2) shows that in Pakistani exposed individuals, the values of total cholesterol were 3.14 times higher compared to Cameroonian exposed individuals. The same observation was made for LDL (Pakistani values were 3.23 times higher).

### 3.4. Biochemical Tests: Hepatic Function

Table 4 depicts the hepatic function in terms of biochemical tests. AST, ALT and LDH were noticeably raised (*p* < 0.05) in both population groups, while Gamma glutamyl transferase (γGT) was statistically different in the Cameroonian group only. Logistic regression (Figure 2) shows that ALP was 4 times and bilirubin was 3.06 times higher in the Pakistani than in the Cameroonian group.

### 3.5. Biochemical Tests: Pancreatic and Renal Function

**Figure 3:**
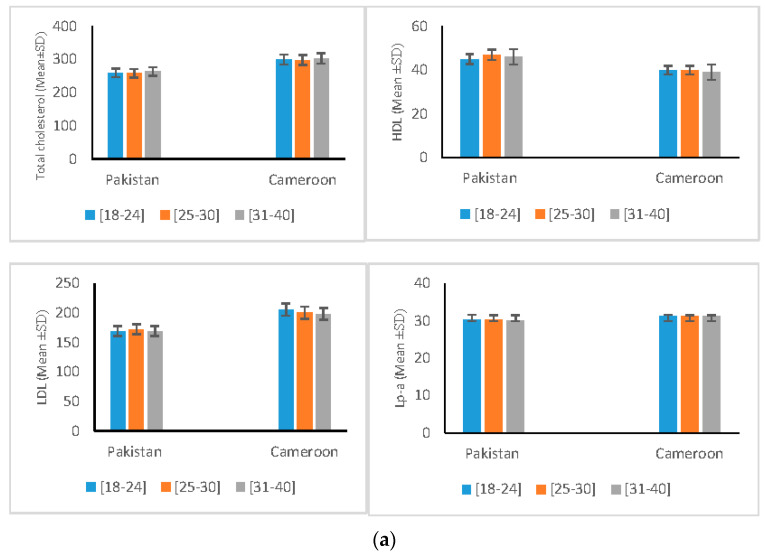

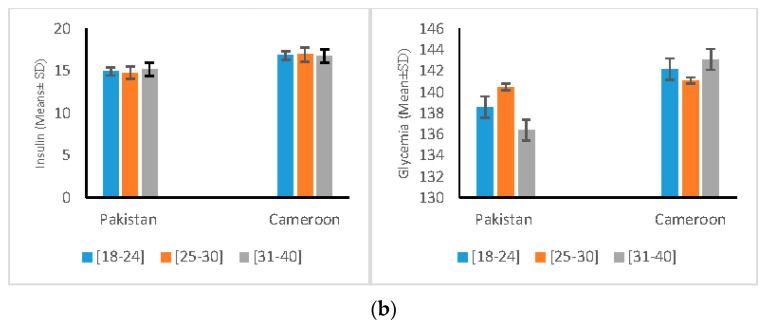

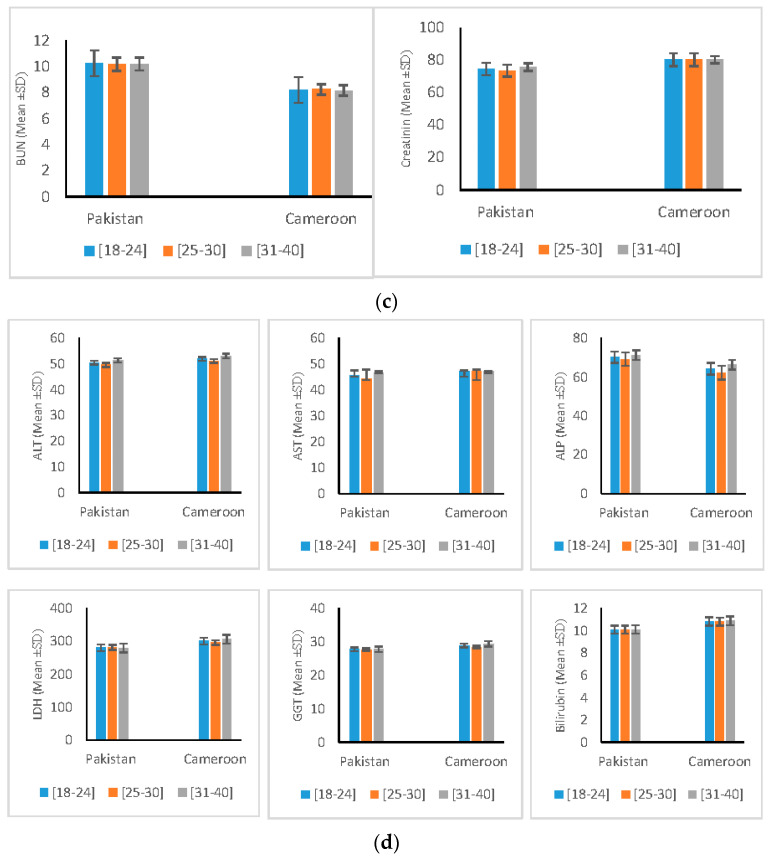
Variation of Biochemical Profile according to age groups, (**a**) Variation of Lipid Profile Parameters according to age group, (**b**) Variation of Diabetic Related Variables Stratified according to age group, (**c**) Variation of Kidney Function Parameters according to age group, (**d**) Variation of Hepatic Function Profile *p* according to age group.

All the studied parameters for pancreatic and renal function (Table 5) show the predisposition and risk of metabolic dysregulation in OPPs exposed groups. No differences in the extent of metabolic dysregulation were observed with regard to gender (in male and female). Additionally, no significant differences in the metabolic dysregulation were noted in any age groups of the two populations (Figure 3).

## 4. Discussion

Use of pesticides has emerged as a major a public health problem globally. Mortality and diseases due to pesticide toxicity have been overwhelmingly documented in the literature [21,22,23,24,25]. More than 80% of insecticides/pesticides used worldwide are organophosphates compounds [26]. In agricultural practice, pesticides are usually applied in combinations and not singly. Organophosphates pesticides are usually used in combination with different OPPs for better pesticide effect. An accumulating number of studies have implicated mixtures of organophosphates in development of chronic ailments as a result of acute or prolonged exposure [27,28,29,30,31]. Metabolic dysregulation has been reported earlier for OPPs as a toxic manifestation but mainly for acute OPPs toxicity. Albeit, long-term OPPs exposure and its possible effects on metabolic homeostasis has been scarcely explored. The aim of the current study was therefore to assess the association of long-term human exposure to OPPs and metabolic disorder in the two population cohorts.

The present study showed the presence of different OPPs in the studied populations, three in Cameroon (Malaoxon, Parathion, and Chlorpyrifos) and two in Pakistan (Malaoxon and Chlorpyrifos). An overt inhibition of RBC-AChE (54.54% in Cameroon and 62.50% in Pakistan) was found, which is considered evidence for OPPs exposure. Decrease of AChE activity is a main characteristic feature and an essential biological marker of OPPs exposure as noted by previous studies [10,32,33]. In fact, organophosphorus compounds can potentially bind to cholinesterase and block their hydrolysis, leading to their rapid accumulation resulting in neurological symptoms (loss of coordination, convulsions, paralysis and possibly death) [34].

Concerning metabolic-related biochemical variables in exposed individuals (with MetS and without MetS), our study revealed a statistically significant increase of LDL, lipoparticles, Creatinine, AST and ALT, and decreased HDL compared to unexposed participants in both countries. The study also observed that exposed people had higher levels of insulin and blood sugar in the Pakistani population. The metabolic dysregulations were more evident in the exposed group with MetS. These metabolic imbalances may be due to the fact that chronic exposure to OPPs may lead to endocrine disruptions, which in turn deregulate enzymes of several metabolic pathways [14,35]. An increase in all these metabolic-related variables (LDL, Lpa, glucose, BMI and blood pressure) has been linked with heightened risk of cardiovascular disease (CVD) and metabolic diseases such as type 2 diabetes. These results support a growing number of studies that have reported that exposure to pesticides such as OPPs would result in the increased blood glucose levels, insulin resistance, and dyslipidemias that are precursors of cardio–metabolic disorders, albeit with conflicting findings [15,36,37,38,39,40,41,42,43,44]. No effect on glucose metabolism after OPPs exposure has also been reported [45]. In the present study, a significant increase in parameters of renal function (creatinine and BUN) and liver function (ALT, AST, LDH, ALP and Bilirubin) was observed in OPPs-exposed subjects in both countries. This increase may be explained by one of two phenomena: the first is the direct toxic effect due to the accumulation of low-dose OPPs over a long period of exposure; the second is that induced type 2 diabetes (T2D) and/or the direct consequences of T2D may lead to the progressive onset of MetS, nonalcoholic fatty liver disease and renal failure [46,47]. The logistic regression model applied to the Pakistani and Cameroonian groups revealed that Pakistani exposed subjects were more likely to develop metabolic diseases compared to other groups. This difference may be due to the fact that the two populations were subject to different environmental conditions (types of pesticides, exposure level, exposure time); the tolerance threshold for toxicity is also different due to genetic differences in the two populations [48,49,50,51].

Although the use and presence of OPPs have been confirmed in the studied groups, certain potential confounding factors such as diet could not be addressed and may constitute a limiting factor of the study.

## 5. Conclusions

This study showed that exposure to a mixture of OPPs significantly affected the metabolic system and may have led to metabolic syndrome in both populations. It is suggested that more randomized controlled studies using larger sample sizes, genetically distinct different cohorts and better exposure monitoring are still needed to ascertain the role of OPPs exposure in initiating metabolic disorders.

## Figures and Tables

**Table 1:** Socio-demographic and Anthropometric data of our study population.

Groups	Pakistan		Cameroon	
	Exposed	Non-Exposed	Exposed	Non-Exposed
Age (Mean ± SD)	28.22 ± 10.12	27.80 ± 10.40	26.57 ± 8.74	26.31 ± 9.07
	N (%)	N (%)	N (%)	**N** (**%**)
Gender				
Male	154 (70)	58 (64.44)	104 (47.27)	45 (47.37)
Female	66 (30)	32 (35.56)	116 (52.73)	50 (52.63)
BMI, frequency				
Underweight	10 (4.55)	4 (4.44)	8 (3.63)	5 (5.26)
Normal range	35 (15.91)	11 (12.22)	37 (16.91)	15 (15.79)
Overweight	89 (40.45)	45 (50)	85 (38.64)	41 (43.16)
Obese	86 (39.09)	30 (33.34)	90 (40.91)	34 (35.79)
High Blood Pressure: mm Hg				
Yes	137 (62.27)	56 (62.22)	168 (76.36)	65 (68.42)
No	83 (37.73)	34 (37.78)	52 (23.64)	30 (31.58)
Smoking				
Yes	48 (21.82)	11 (12.22)	10 (4.54)	4 (4.21)
No	172 (78.18)	79 (87.78)	210 (95.46)	91 (95.79)

N = total number of samples, Results are in mean ± SD.

**Table 2:** Variation of Cholinergic/PON1 enzymes of OPPs exposed and nonexposed groups in two populations.

Parameters	Pakistan			Cameroon		
	Exposed N = 220	Unexposed N = 90	*p*-Value	Exposed N = 220	Unexposed N = 95	*p*-Value
RBC-AChE mU/µmol Hb	0.13 ± 0.04 ^b^	0.30 ± 0.07 ^d^	˂0.01	0.10 ± 0.06 ^a^	0.23 ± 0.10 ^c^	˂0.01
BChE µmol/l/min	0.021 ± 0.010 ^b^	0.031 ± 0.007 ^c^	˂0.05	0.018 ± 0.011 ^a^	0.023 ± 0.010 ^bc^	˂0.05
PON1 ng/mL	5.49 ± 1.01 ^b^	15.11 ± 4.20 ^d^	˂0.01	2.86 ± 1.47 ^a^	11.83 ± 2.17 ^c^	˂0.01

^a,b,c,d^ = statistical difference of post-hoc test (Anova one way). Results are in mean ± SD; the result are statistically significant when *p* ≤ 0.05).

**Table 3:** Lipid profile of the OPPs exposed and nonexposed groups in two populations.

	Pakistan				Cameroon			
	Exposed and MetS N = 100	Non-Exposed and MetS N = 90	Exposed but No MetS N = 120	*p* Value	Exposed and MetS N = 108	Non-Exposed and MetS N = 95	Exposed but No MetS N = 112	*p* Value
Tchosl * (mg/dL)	260 ± 60 ^b^	200 ± 51 ^a^	200 ± 54 ^a^	<0.001	300 ± 58 ^b^	251 ± 62 ^a^	263 ± 80 ^a^	<0.001
HDL *# (mg/dL)	46 ± 6 ^a^	64 ± 9 ^c^	58 ± 12 ^a^	<0.001	39 ± 4 ^a^	48 ± 11 ^a^	47 ± 17 ^a^	>0.05
LDL * (mg/dL)	170 ± 7 ^b^	130 ± 10 ^a^	136 ± 8 ^a^	<0.001	201 ± 64 ^b^	184 ± 39 ^a^	180 ± 54 ^a^	<0.05
Lp(a) * (mg/dL)	30.12 ± 2.30 ^c^	15.31 ± 2.49 ^a^	22.22 ± 5.23 ^b^	<0.01	31.25 ± 7.20 ^b^	10.05 ± 6.30 ^a^	30.04 ± 6.31 ^b^	<0.001

N = total number of samples, Tchosl = total cholesterol; HDL = high density lipoprotein; LDL = low density lipoprotein; Lp(a) = Lipoparticule a. ^a,b,c^ = statistical difference of post hoc test (Anova one way). Results are in mean ± SD. * statistically significant (*p* ≤ 0.05), # only Cameroonian group is statistically significant (*p* ≤ 0.05).

**Table 4:** Hepatic function variables in OPPs exposed and nonexposed groups in two populations.

Pakistan					Cameroon			
	Exposed and MetS N = 100	Non-Exposed and MetS N = 90	Exposed but No MetS N = 120	*p* Value	Exposed and MetS N = 108	Non-Exposedand MetS N = 95	Exposed but No MetS N = 112	*p* Value
AST * (IU/L)	45.52 ± 30.24 ^b^	26.09 ± 7.90 ^a^	44.80 ± 31.37 ^b^	<0.01	47.20 ± 16.10 ^b^	26.50 ± 9.72 ^a^	50.61 ± 20.01 ^b^	<0.01
ALT * (IU/L)	50.44 ± 33.45 ^b^	26.55 ± 5.50 ^a^	50.17 ± 30.06 ^b^	<0.01	52.02 ± 10.28 ^b^	30.30 ± 13.56 ^a^	55.46 ± 14.43 ^b^	<0.01
LDH * (IU/L)	280.34 ± 60.80 ^b^	208.61 ± 59.14 ^a^	264.19 ± 75.50 ^b^	<0.01	300.82 ± 40.20 ^b^	220.54 ± 24.61 ^a^	262.40 ± 38.17 ^ab^	<0.01
γGT # (IU/L)	27.70 ± 9.84 ^a^	27.69 ± 10.10 ^a^	29.79 ± 10.87 ^a^	>0.05	28.91 ± 10.40 ^a^	30.50 ± 16.90 ^a^	35.80 ± 17.27 ^b^	<0.05
ALP (IU/L)	70.14 ± 23.14 ^a^	68.79 ± 16.20 ^a^	67.02 ± 20.20 ^a^	>0.05	64.23 ± 13.20 ^a^	60.82 ± 14.78 ^a^	60.52 ± 14.12 ^a^	>0.05
D Bil (mg/L)	4.19 ± 2.11 ^a^	3.61 ± 0.56 ^a^	4.19 ± 2.30 ^a^	>0.05	4.38 ± 1.49 ^a^	4.57 ± 1.70 ^a^	4.27 ± 1.81 ^a^	>0.05
T Bil (mg/L)	10.09 ± 4.90 ^a^	10.40 ± 1.92 ^a^	9.59 ± 4.90 ^a^	>0.05	10.83 ± 3.68 ^a^	11.07 ± 2.83 ^a^	11.44 ± 3.27 ^a^	>0.05

N = total number of samples, AST = aspartate transaminase; ALT = alanine transaminase; LDH = lactate deshydrogenase; GGT = gamma-glutamyl transferase; ALP = alkalin phosphatase; DBil = direct bilirubin; TBil = total bilirubin. Results are mean ± SD. * statistically significant (*p* ≤ 0.05). # only Cameroonian group is statistically significant (*p* ≤ 0.05). ^a,b^ = statistical difference of post hoc test (Anova one way).

**Table 5:** Pancreatic and renal function related variables in two population groups.

	Pakistan				Cameroon			
	Exposed and MetS N = 100	Non-Exposed and MetS N = 90	Exposed but No MetS N = 120	*p* Value	Exposed and MetS N = 108	Non-Exposed and MetS N = 95	Exposed but No MetS N = 112	*p* Value
Glucose * (mg/dL)	138.50 ± 12.22 ^b^	125.25 ± 10.50 ^a^	123.56 ± 8.81 ^a^	<0.05	142.12 ± 1.51 ^a^	135.02 ± 1.18 ^a^	131.01 ± 2.12 ^a^	>0.05
Insulin * (µIU/mL)	14.97 ± 2.71 ^a^	12.46 ± 1.50 ^a^	11.57 ± 3.64 ^a^	<0.05	16.91 ± 0.70 ^a^	17.35 ± 0.73 ^a^	17.10 ± 0.66 ^a^	>0.05
BUN *# (mmol/L)	10.20 ± 3.90 ^b^	6.14 ± 1.3 ^a^	9.12 ± 4.18 ^b^	<0.01	8.20 ± 2.70 ^a^	9.06 ± 1.20 ^a^	8.15 ± 1.99 ^a^	>0.05
Creatinine * (µmol/L)	74.49 ± 20.14 ^b^	60.40 ± 14.70 ^a^	70.28 ± 20.33 ^b^	<0.05	80.20 ± 20.01 ^b^	65.23 ± 12.02 ^a^	78.15 ± 18.80 ^b^	<0.05

BUN = Blood urea nitrogen, N = total number of samples, ^a,b^ = statistical difference of post hoc test (Anova one way). * statistically significant at *p* ≤ 0.05. # not significant in Cameroon group.

## Data Availability

The data presented in this study are available on request from the corresponding author.
